# Acute lymphoblastic leukemia first presenting with ascites: A case report

**DOI:** 10.1097/MD.0000000000041175

**Published:** 2025-01-03

**Authors:** Fengxue Yu, Zhen Tao, Cong Cheng, Hongxia Wei, Rentian Cai

**Affiliations:** a Department of Radiotherapy, The Second Hospital of Nanjing, Affiliated to Nanjing University of Chinese Medicine, Nanjing, China; b Department of Infectious Disease, Nanjing First Hospital, Nanjing Medical University, Nanjing, China; c Department of Infectious Disease, The Second Hospital of Nanjing, Affiliated to Nanjing University of Chinese Medicine, Nanjing, China.

**Keywords:** acute lymphoblastic leukemia, ascites, case report, first presenting, leukemia

## Abstract

**Rationale:**

Acute lymphoblastic leukemia (ALL) is known for its tendency to present with extramedullary infiltrates, commonly affecting lymph nodes, the spleen, the central nervous system, the orbit, skin, and testes. Less commonly, leukemia can infiltrate unusual sites such as the cervix, gastrointestinal tract, lungs, and thyroid gland. However, it is exceedingly rare for leukemia to present with ascites as the initial symptom. By reporting this rare case, the study seeks to contribute to a better understanding of ALL’s varied presentations and improve early detection and management strategies, ultimately enhancing patient outcomes.

**Patient concerns:**

A 30-year-old male was admitted to the hospital with the primary complaint of ascites.

**Diagnosis:**

ALL.

**Interventions:**

The patient was initially diagnosed with suppurative peritonitis through abdominal puncture. The abdominal computed tomography scan revealed ileocecal lesions, which were thought to be the source of the purulent peritonitis. Despite receiving antibacterial therapy and undergoing peritoneal effusion drainage, the treatment proved ineffective. The patient chose to discontinue hospitalization and was discharged on June 22. On August 24, he was readmitted to Nanjing First Hospital. A follow-up abdominal computed tomography scan revealed multiple enlarged lymph nodes in the abdomen, raising suspicion of a hematological malignancy. Bone marrow cytology subsequently confirmed the diagnosis of ALL.

**Outcomes:**

The patient was admitted to the hematology department for further treatment. Unfortunately, despite intervention, the patient passed away.

**Lessons:**

Although it is rare for ALL to present initially with ascites, clinicians should consider leukemia as a potential diagnosis in patients presenting with ascites, particularly when conventional treatments for other diagnoses are ineffective.

## 1. Introduction

Leukemia frequently presents with extramedullary infiltrates, which are commonly observed in lymph nodes, the spleen, the central nervous system, the orbit, the skin, and the testes. Less typical sites of infiltration include the cervix, gastrointestinal tract, lungs, and thyroid gland. However, it is exceedingly rare for leukemia to manifest with ascites as the primary symptom.

This article reports a case of acute lymphoblastic leukemia (ALL) where ascites was the initial presentation.

## 2. Case presentation

### 2.1. Ethics statement and patient consent for publication

Ethical approval was waived by the ethics committee, as this article is a case report and all tests performed were routine examinations for the disease during that period. The written informed consent was secured from the patient.

A 30-year-old man was admitted to our hospital on June 6, 2020, presenting with abdominal distension that had developed over the preceding 2 weeks. Initially, the patient noted abdominal distension without any accompanying symptoms such as pain, diarrhea, nausea, vomiting, or fever and, thus, did not seek immediate medical attention. As the distension worsened, he sought evaluation at our outpatient department. An abdominal ultrasound revealed ascites, leading to his admission.

On physical examination, the patient’s temperature was 38.2 °C, with clear consciousness and overall good condition. Heart and lung examinations revealed no abnormalities. Abdominal examination showed distension with slight tenderness in the right lower quadrant, positive moving dullness, and no muscle rigidity or rebound tenderness.

The differential diagnosis included tuberculous peritonitis, purulent peritonitis, and cirrhosis-associated ascites. Laboratory tests at admission revealed the following.

Complete blood count: White blood cell count of 11.7 × 10^9^/L, neutrophil percentage of 83.2%, lymphocyte percentage of 11.7%, hemoglobin of 162 g/L, and platelet count of 423 × 10^9^/L.

Biochemistry: Alanine aminotransferase of 40.0 U/L, aspartate aminotransferase of 33.0 U/L, phosphocreatine kinase of 60.0 U/L, albumin of 39.8 g/L, glutamyl transpeptidase of 16.0 U/L, total bilirubin of 6.4 µmol/L, direct bilirubin of 1.7 µmol/L, C-reactive protein of 10.2 mg/L, and procalcitonin of 0.077 ng/L.

Infection screenings for hepatitis B virus, hepatitis C virus, human immunodeficiency virus, and syphilis were negative. Liver stiffness testing was normal. Abdominal computed tomography (CT) showed thickening of the small intestine and ileocecal wall, disorganization of intestinal structure, thickening of the greater omentum, and an increased number of lymph nodes in the abdominal cavity, suggesting peritonitis.

### 2.2. Diagnostic procedures and initial management

An abdominal puncture was performed, yielding turbid ascitic fluid with a leukocyte count of 139 × 10^9^/L, predominantly multinucleated cells (98%). Biochemical analysis of the ascitic fluid revealed: albumin of 33.0 g/L, lactate dehydrogenase of 3554.0 U/L, total protein of 48.8 g/L, glucose of 0.07 mmol/L, chloride of 105.6 mmol/L, and adenosine deaminase of 39.5 U/L. Interferon levels in ascitic fluid were normal, and cultures were negative. The initial diagnosis was suppurative peritonitis. Treatment with cefotaxime and sulbactam (4.5 g, intravenous drip, twice a day) was initiated to target common gram-negative bacilli of intestinal origin.

**Figure 1.**
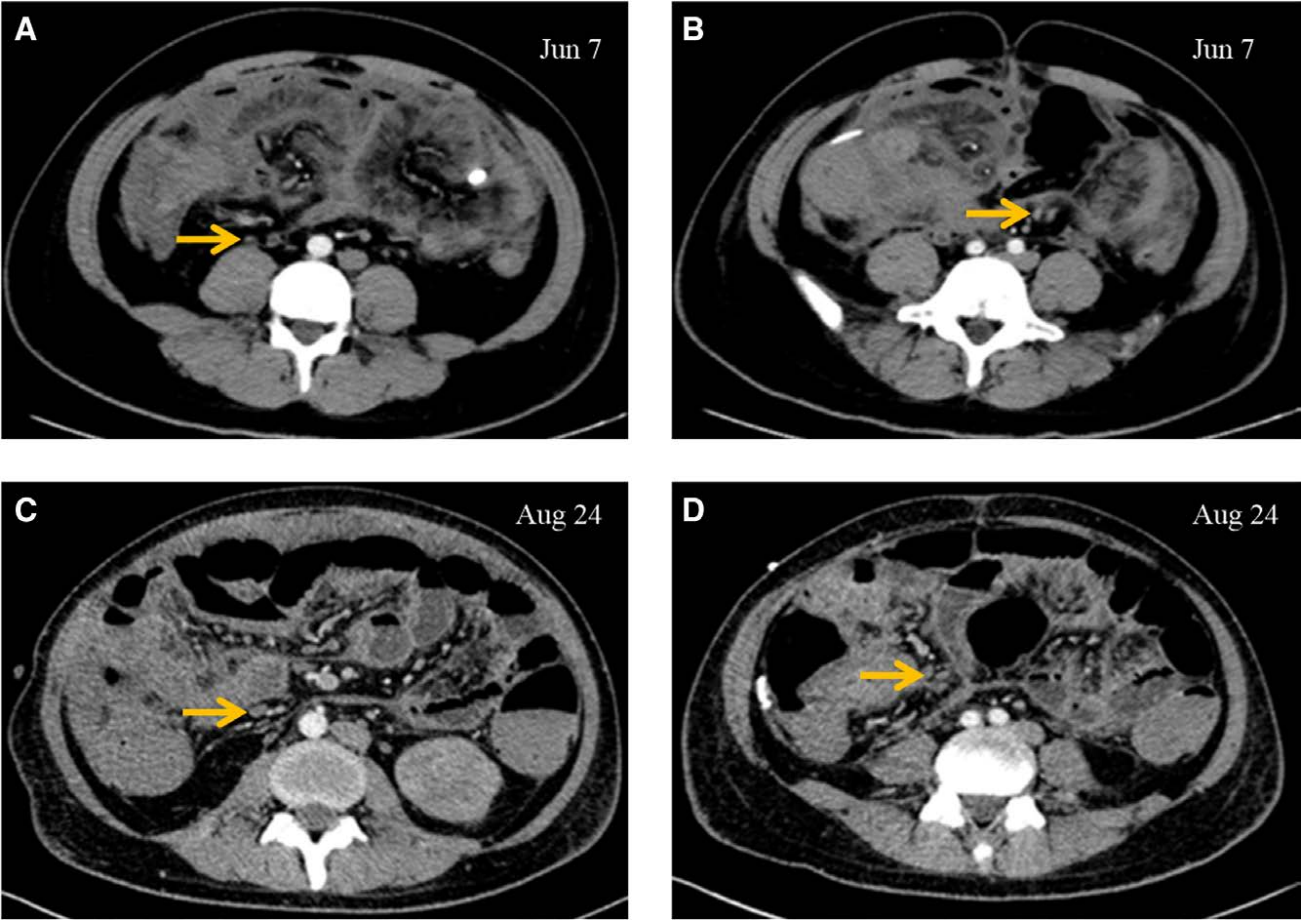
Abdominal computed tomography (CT) scan with contrast abdominal CT scan with contrast on June 7 (A and B) revealed an increase in the size and number of abdominal lymph nodes, which were even larger on August 24 (C and D).

Further evaluations, including purified protein derivative and T-spot tuberculosis tests, were negative, and normal interferon levels in ascitic fluid did not support tuberculous peritonitis. Continued investigation revealed ileocecal lesions (Fig. 1A and 1B) that were suspected to cause purulent peritonitis. The gastrointestinal surgeon noted the presence of lesions in the ileocecal area and potential appendix perforation but recommended against surgery. Instead, they advised continued antibacterial therapy.

Despite the addition of etimicin (0.3 g, intravenous drip, once a day) on June 15, the patient’s condition did not improve. The antibiotic regimen was adjusted to imipenem and cilastatin sodium (2.0 g, intravenous drip, once every 8 hours) on the same date. While the patient’s fever resolved, ascites persisted. Nutritional support was provided, and additional ascitic fluid analyses were performed, consistently showing no tumor cells or evidence of tuberculosis. The patient and his family chose to discharge him against medical advice on June 22.

**Figure 2.**
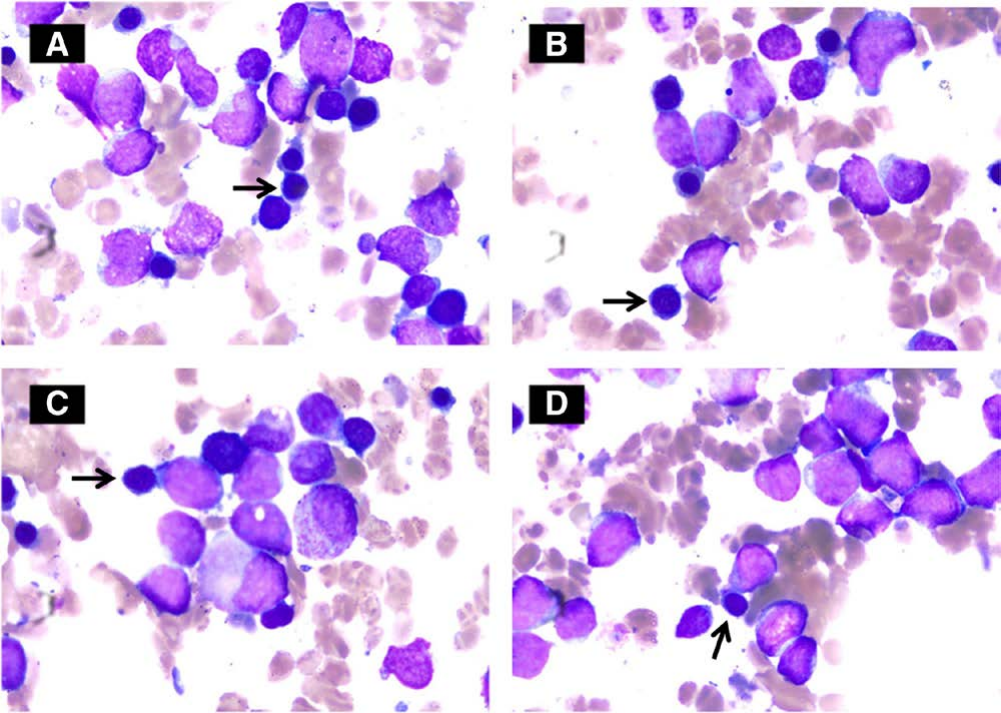
Bone marrow cytology: acute lymphoblastic leukemia. Primitive lymphocytes and naive lymphocytes accounted for 40.2% and 10.4% of leukocytes, respectively.

After discharge, the patient continued to experience ascites and fever but did not seek further treatment. Despite recommendations for further evaluation at a larger facility, the patient refused additional care. On August 24, 2020, he was readmitted to Nanjing First Hospital. A repeat abdominal CT showed multiple enlarged lymph nodes in the abdomen, raising suspicion of a hematological malignancy (Fig. 1C and 1D). Bone marrow aspiration confirmed the diagnosis of ALL (Fig. 2).

## 3. Differential diagnosis

### 3.1. Tuberculous peritonitis

The patient, a young male with fever and ascites, underwent several tests to rule out tuberculous peritonitis. Acid-fast staining of the ascitic fluid was negative, and the concentration of interferon-γ in the ascitic fluid was not elevated. In addition, the peripheral blood T-spot tuberculosis test was negative. These findings collectively excluded tuberculous peritonitis.

### 3.2. Purulent peritonitis

The patient presented with fever and ascites. Routine analysis of the ascitic fluid revealed a significant increase in white blood cells and a predominance of neutrophils. Although bacterial cultures were inconclusive, the patient’s fever subsided and the white blood cell count in the ascitic fluid decreased following antibacterial treatment. These clinical responses support the diagnosis of purulent peritonitis.

### 3.3. Cirrhosis-associated ascites

The patient, a young male, underwent liver stiffness measurement using fibro touch, which indicated normal liver hardness. In addition, abdominal CT did not show signs of cirrhosis. Therefore, cirrhosis-associated ascites was excluded.

## 4. Outcome and follow-up

The patient was admitted to the hematology department for further treatment. Unfortunately, despite intervention, the patient’s condition deteriorated, and he eventually passed away.

## 5. Discussion

ALL is a prevalent hematological malignancy, typically characterized by clinical manifestations such as fever, hemorrhage, and enlargement of the liver, spleen, and lymph nodes. However, ascites as the initial presenting symptom of ALL is exceedingly rare. In the case we present, the patient experienced a rapid increase in ascites, with intermittent fever that was not perceived by the patient, possibly due to an insufficient sensitivity to fever.

Initially, diagnostic evaluations suggested that ileocecal lesions were responsible for suppurative peritonitis. Despite antibacterial therapy, the patient’s condition did not improve, indicating that the underlying cause was not addressed. As the disease progressed, the patient was eventually diagnosed with ALL.

The literature contains few reports of leukemia presenting with ascites as the primary symptom. For example, a 57-year-old Japanese female presented with pleural effusion, pericardial effusion, and ascites but no notable superficial lymphadenopathy. Cytological analysis of the ascitic fluid revealed numerous lymphomatous cells, leading to a diagnosis of CD4^−^/CD8^+^ adult T-cell leukemia/lymphoma. Despite treatment with a vincristine/cyclophosphamide/doxorubicin/prednisolone regimen, the patient’s condition rapidly deteriorated, and she died within a month.^[1]^

Another case involved a 77-year-old woman with a history of colorectal cancer who presented with abdominal discomfort, ascites, pleural effusion, and splenomegaly. Bone marrow examination ultimately diagnosed her with T-cell prolymphocytic leukemia. She underwent 3 courses of fludarabine therapy without a favorable response.^[2]^

A 27-year-old male presented with ascites, confusion, and pancytopenia. Bone marrow aspiration revealed early pre-B cell leukemia, and after 5 courses of chemotherapy, the patient achieved remission by August 2006.^[3]^

In 1991, a 67-year-old man presented with ascites and pleural effusion. Adult T-cell leukemia was diagnosed based on specific surface markers for T lymphocytes in pleural effusion and ascitic fluid. Although chemotherapy led to the resolution of ascites and pleural effusion within 2 weeks, the patient succumbed to pneumonia during subsequent treatment.^[4]^

Ascites, or the accumulation of fluid in the abdominal cavity, can be an unusual yet significant manifestation in various forms of leukemia. For instance, both hairy cell leukemia and chronic lymphocytic leukemia have been documented to present with massive ascites. Arora et al^[5]^ and Kayal et al^[6]^ reported cases of hairy cell leukemia with concurrent ascites, highlighting the rarity of this presentation in such cases. Similarly, Gogia et al^[7]^ and Huang et al^[8]^ described chronic lymphocytic leukemia patients presenting with massive ascites, with the latter diagnosed through gene rearrangement assays. This symptom can also manifest in other leukemia types such as acute monocytic leukemia, which was observed to have associated gastric infiltration and ascites as reported by Domingo-Domenech et al.^[9,10]^ The phenomenon of leukemic ascites underscores the diverse presentations of hematological malignancies and illustrates the need for awareness of these atypical manifestations in clinical practice.^[11–13]^

Ascites as the first symptom of ALL remains an extremely rare presentation, often leading to delayed diagnosis. Early recognition and accurate diagnosis depend on heightened awareness among clinicians. Essential diagnostic steps include precise cytological examination of ascitic fluid and timely bone marrow aspiration.

## 6. Learning points

Leukemia can occasionally present with atypical symptoms such as ascites though this is uncommon. In this case, a young man presenting with ascites was initially misdiagnosed with a different condition. When initial treatments proved ineffective, further testing revealed the correct diagnosis of ALL. This case underscores the importance of considering leukemia in the differential diagnosis when encountering unusual presentations such as ascites to ensure timely and accurate treatment.

## 7. Limitations

The limitations of this study include the rare nature of ascites as an initial symptom of ALL, making it difficult to generalize the findings. The case is based on a retrospective report of a single patient, limiting its statistical power and introducing potential bias. In addition, the patient’s delayed return to treatment after initial discharge, misdiagnosis at the outset, and the study’s single-center design further constrain the generalizability of the results. Finally, the patient’s rapid deterioration and poor prognosis limit the ability to assess the effectiveness of treatments for this unusual presentation of ALL.

## Acknowledgments

The authors are grateful to the patient for allowing us to document their medical history.

## Author contributions

**Data curation:** Fengxue Yu, Zhen Tao

**Formal analysis:** Fengxue Yu

**Investigation:** Fengxue Yu

**Software:** Fengxue Yu, Cong Cheng

**Writing – original draft:** Fengxue Yu

**Methodology:** Cong Cheng, Hongxia Wei, Rentian Cai

**Conceptualization:** Hongxia Wei, Rentian Cai

**Writing – review & editing:** Hongxia Wei, Rentian Cai
